# Aligning social media design with adolescent brain development: rationale and recommendations

**DOI:** 10.3389/fdgth.2026.1880395

**Published:** 2026-08-04

**Authors:** Yashraj Garg, Jin Siriudomsait, Benjamin Chidiac

**Affiliations:** 1 Department of Psychology, School of the Biological Sciences, University of Cambridge, Cambridge, United Kingdom; 2 Department of Psychiatry, School of Clinical Medicine, University of Cambridge, Cambridge, United Kingdom

**Keywords:** adolescence, digital policy, functional MRI, neurodevelopment, social media, structural MRI

## Abstract

The human brain and its associated faculties develop in a non-linear, asynchronous way across childhood and adolescence. Current digital technology policies, especially those involving social media, have failed to adequately account for this in their design. To maximise efficacy and societal benefit, such policies must be biologically informed. Synthesising across existing neuroscientific evidence, we propose a developmentally staged framework for biologically informed social media design and regulation. If implemented, the resulting recommendations may help to safeguard young people during particular periods of vulnerability while supporting healthy growth and learning opportunities.

## 1 Introduction

Concerns about the effects of social media on adolescent wellbeing have prompted increasingly restrictive policy responses, including age-based access limits and platform bans. In December 2025, Australia implemented a blanket ban on social media for children under 16 (1), while the UK Government has recently announced an equivalent ban for under-16s, with implementation targeted for early 2027 (2). These interventions reflect legitimate concern about developmental vulnerability but implicitly assume developmental risk can be appropriately managed by identifying a single age at which unrestricted social media access becomes acceptable. Regardless of the inevitable implementation challenges, the binary logic underlying such approaches sits at odds with contemporary developmental neuroscience. Adolescent brain development does not proceed through linear or uniform maturation, but through asynchronous, non-linear changes across neural systems involved in reward processing, social evaluation, and cognitive control (3, 4). Consequently, developmental sensitivity fluctuates throughout the entirety of adolescence, suggesting no biologically optimal age at which unrestricted social media exposure becomes appropriate.

A further complication is that social media platforms are not passive channels of exposure. Instead, they are algorithmically curated environments with adaptive recommendation systems that continuously monitor and respond to user behaviour, shaping future content delivery through reinforcement-based feedback loops. With this comes potential for developmental sensitivities to be amplified and exploited without limit. Developmental risk therefore emerges from the engineered interaction between neurodevelopmental stage and algorithmic environment, rather than from exposure alone.

Taken together, the dynamic properties of adolescent neurodevelopment and adaptive recommendation systems point to the need for alignment. Rather than conceptualising social media regulation as a binary question of access, digital platforms themselves should be regarded as adaptive environments whose algorithmic behaviour can be calibrated to changing neurodevelopmental contexts. Graduated exposure, rather than an all-or-nothing approach, offers a biologically informed solution that preserves opportunities to improve digital literacy while reducing harmful exposure during periods of heightened vulnerability. On this basis, we propose a framework of developmentally staged algorithmic design as an alternative to both blanket bans and unrestricted platform access.

## 2 Understanding non-linear and asynchronous adolescent brain development

### 2.1 Asynchronous maturation of brain systems

A central feature of adolescent neurodevelopment is the asynchronous maturation of large-scale brain systems. Primary sensory areas develop quickly in early childhood, while more complex, higher-order association areas take significantly longer and continue to develop into adulthood (5, 6). Subcortical regions implicated in reward processing and salience detection mature around ages 10–15 years, while the prefrontal and frontoparietal networks that regulate these regions continue to develop into the mid-20s (3, 7). This temporal imbalance has been widely described in dual-systems models of adolescence, in which heightened reward sensitivity coexists with still-developing regulatory capacity (7). Of course, this imbalance has important behavioural consequences. Increased responsivity to reward and novelty, combined with immature executive control, biases behaviour towards impulsive reinforcement, particularly in socially salient contexts.

### 2.2 Non-linear trajectories and developmental reorganisation

Adolescent brain development is further characterised by non-linear trajectories in network organisation. Longitudinal analyses of functional connectivity demonstrate that development between ages 14 and 26 involves both “conservative” and “disruptive” modes of change that affect primary sensorimotor and association networks differently (8). These changes reflect a transition from locally segregated networks towards more distributed, integrated systems where advanced higher-order networks play a more influential role. Complementary work shows that global functional network efficiency increases with age while local efficiency and modularity decrease, following non-linear trajectories (9). Late childhood (approximately ages 8–11) also appears to be a period of pronounced structural topological reorganisation, characterised by shifts in network integration and the emergence of connector hubs linking distributed regions (5). These findings suggest the presence of sensitive periods during which different aspects of network architecture become malleable in different ways. Such non-linear dynamics give rise to temporally specific windows of heightened plasticity and vulnerability, underlining the importance of adaptive protection as higher-level cognitive systems gradually come online.

### 2.3 Development of the social brain and affective bias

In parallel with these network-level changes, adolescence involves protracted development of the “social brain”, including medial prefrontal, temporoparietal, and anterior temporal regions (10). Important structural consolidation of these regions begins around age 9 and continues until the early to mid-20s, suggesting no optimal time to introduce risks like social media exposure (11, 12). Developmental asynchrony is also observed between the default mode network—implicated in self-referential thought and social cognition—which shows greatest functional maturation relative to other systems in the first years of life, and the salience network, which facilitates attention control and adaptive behaviour, and develops most from ages 10–15 (13). This temporal offset may contribute to heightened emotional sensitivity, increased self-focus, and slower recovery from social stressors during this period.

In parallel, connectivity between the amygdala and prefrontal cortex undergoes significant reorganisation, transitioning from positive coupling in childhood to more regulatory, inverse coupling in adolescence (14). This shift is associated with improvements in emotional regulation and reduced anxiety but remains incomplete until later adolescence (approximately ages 16–18), with regulatory coupling still stabilising across this period (15). Taken together, these findings indicate that adolescent neurodevelopment involves shifting configurations of social, affective, and regulatory networks that may heighten sensitivity to social feedback and emotional salience: features that are strongly amplified in contemporary social media environments.

## 3 Social media environments as adaptive developmental inputs

### 3.1 Reinforcement learning and reward sensitivity

**Figure 1:**
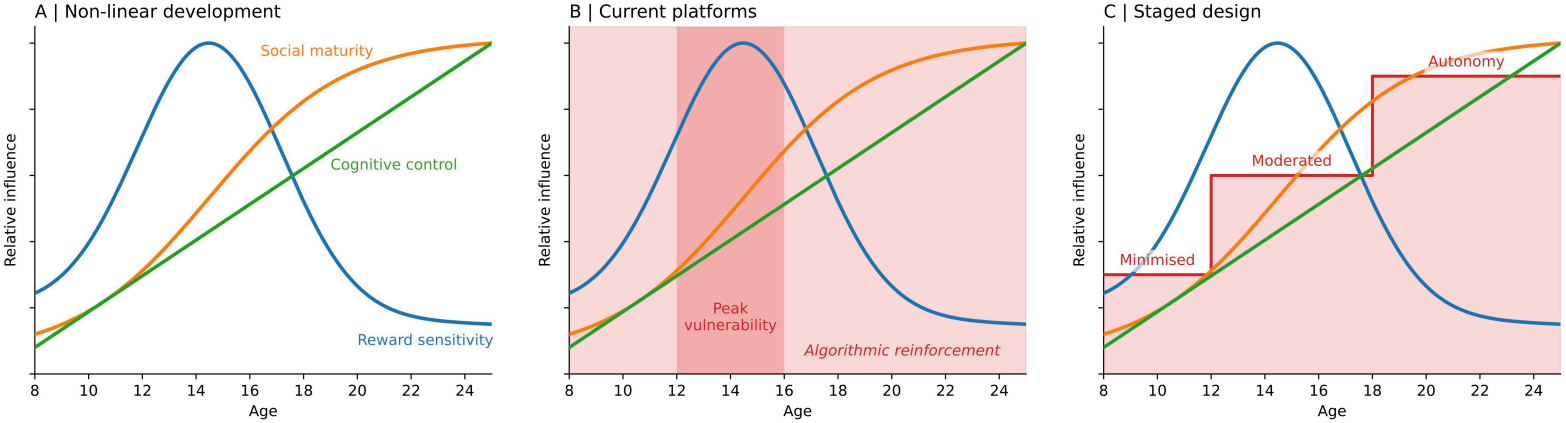
Non-linear adolescent brain development and implications for algorithmic design. **(A)** Developmental trajectories of reward sensitivity, social maturity, and cognitive control illustrate asynchronous and non-linear maturation. **(B)** Current social media platforms deliver continuous, high-intensity reinforcement (red shading), creating a mismatch between peak sensitivity and immature control (darker red). **(C)** Developmentally staged design modulates algorithmic exposure across neural maturation, minimising risk while preserving opportunity. The trajectories shown are schematic and are not derived from a single empirical dataset.

As illustrated in Figure 1, contemporary social media platforms deliver constant reinforcement that may disproportionately engage neural systems undergoing non-linear development, particularly during periods of peak vulnerability. These platforms are designed to be engagement-maximising architectures (16, 17), employing algorithmic personalisation (17, 18), intermittent reinforcement schedules (19), and continuous behavioural optimisation mechanisms (20). Rather than operating as passive communication tools, they dynamically modify presented information according to previous user interactions, thereby reinforcing behaviours associated with prolonged engagement (21, 22). Features such as personalised recommendations, likes, notifications, infinite scroll, and algorithmically prioritised content (23) all engage dopaminergic reward systems while simultaneously shaping future delivery. Stronger responses to certain content lead to increased frequency and intensity of presentation in the future, creating a self-amplifying cycle that can be particularly dangerous during adolescence.

### 3.2 Continuous and personalised exposure

As continuously adapting systems, the influence of social media environments is not a consistent force. Depending on developmental stage and associated sensitivities, these environments will respond to and shape behaviour in markedly different ways. We have created a technology that has produced an evolutionary mismatch: an amplified system of social engagement that can change at a rate far faster than our biology can develop (24). While this has clear potential to exploit our developmental vulnerabilities, it is also equally capable of serving as an agile, proactive support system for healthy growth. This mismatch does not need to be inherently harmful, but its effects depend on how we choose to align exposure with developmental processes.

### 3.3 The mismatch between neuroscience and policy

Despite increasing concern about adolescent mental health, policy responses have largely adopted binary frameworks, such as age-based bans or strict usage limits. These approaches implicitly assume that developmental risk can be managed by identifying a threshold at which exposure becomes acceptable. However, developmental neuroscience does not identify a single age during adolescence at which unrestricted exposure can be assumed to become appropriate.

Binary regulatory approaches risk creating a mismatch between developmental need and environmental design. Blanket bans may delay opportunities to develop digital literacy and healthy usage habits within supportive environments, whereas unrestricted access exposes adolescents to highly optimised reinforcement systems during periods of peak sensitivity. In both cases, current policy fails to account for the dynamic interplay between developmental stage and environmental input.

## 4 Practical recommendations

### 4.1 A framework for developmentally staged algorithmic design

In light of neurodevelopmental evidence, we propose an alternative to current social media policy approaches: developmentally staged algorithmic design. Presented in Table 1, this framework describes how phased restriction of algorithmic function can be used to facilitate graduated, developmentally supportive exposure in line with the changing properties of the young brain. This proposed, evidence-informed framework focuses on safeguarding developmental vulnerabilities while maximising healthy learning opportunities and comprises three stages: late childhood, early-mid adolescence and mid-late adolescence.

**Table 1:** Developmentally staged algorithmic design framework.

Developmental stage	Approx. age	Neural profile	Algorithmic design features	Policy objective
Late childhood	8–11	High plasticity; large-scale network reorganisation begins; emerging social cognition	Very minimal personalisation; hidden or de-emphasised engagement feedback/metrics; no infinite scroll; slower content refresh	Minimise early reinforcement loops and habitual engagement
Early-mid adolescence	12–15	Peak reward and social sensitivity; immature cognitive control; heightened affective reactivity	Reduced visibility of likes/shares; friction in scrolling and autoplay; capped recommendation intensity; slower algorithmic adaptation	Reduce vulnerability during peak sensitivity
Mid-late adolescence	16–18+	Strengthening frontoparietal control; improving emotion regulation; stabilising social cognition networks	Gradual increase in personalisation; transparent, modifiable recommendation logic; user-controlled settings; digital self-regulation tools	Support autonomy, self-regulation and informed decision making

Algorithmic features can be aligned with developmental changes in neural systems across adolescence. Late childhood is characterised by heightened plasticity and network reorganisation, suggesting the need for limited personalisation and restricted functionality. Early-mid adolescence involves peak reward and social sensitivity alongside immature cognitive control, indicating the importance of dampening feedback and limiting rapid reinforcement. In later adolescence, improving cognitive-affective regulatory capacity supports the gradual introduction of autonomy and user control.

In late childhood (approximately ages 8–11), a period characterised by widespread network reorganisation and heightened plasticity, platforms should heavily limit personalisation, slow content refresh rates and reduce the salience of engagement metrics. This may help prevent early reinforcement of habitual engagement patterns while supporting gradual familiarisation with digital social environments.

During early and mid-adolescence (ages 12–15), when reward and social sensitivity peak, algorithm regulation must aim to dampen reinforcement loops. This does not just refer to the reinforcement of user engagement, but also algorithmic reinforcement of hyper-salient content presentation. Practically, this should involve reduced frequency or visibility of feedback signals, friction for infinite scrolling, and restricted recommendation intensity.

As cognitive control and emotional regulation improve into later adolescence (ages 16–18+), greater user autonomy can be introduced. This should be paired with transparent recommendation systems, user-controlled settings, and tools to support self-regulation. Consistent with evidence that adolescence represents a period of both vulnerability and opportunity (25), this approach may support adaptive learning and resilience, helping to foster healthier usage patterns into adulthood.

An essential goal of developmentally staged algorithmic design is to create opportunities to enhance digital literacy from early on. Rather than a feared inevitability, early social media exposure may in fact become a healthy growth experience, but only if enabled in the correct way (26). By tailoring educational objectives to underlying neurodevelopment, foundational competencies can be established and built on in a manner that demands less of young users themselves. During late childhood, key objectives should include understanding basic online safety, recognising persuasive design features, and developing healthy engagement patterns. Over early-mid adolescence, emphasis should be placed on algorithmic awareness, critical evaluation of online information, and strategies for sensitivity management and self-control. By later adolescence, the educational focus should be predominantly geared towards independent self-regulation, informed decision-making, and continued critical skill development (26). By shaping the nature and timing of social media exposure in a more intentional, biologically principled way, it might be possible to maximise its benefits while better controlling its risks.

### 4.2 Limitations and future directions

The translation of developmental neuroscience into policy remains challenging. Evidence linking social media use to specific mental health outcomes is largely correlational and heterogeneous, with small average effect sizes and substantial individual variation (27). As such, causal mechanisms remain incompletely understood. In addition, individual differences in developmental trajectories complicate attempts to define universal intervention points (3). While developmental staging provides a more nuanced framework than binary age thresholds, it still relies on imperfect proxies for neural maturity that do not represent all individuals equally.

Practical and ethical challenges also arise. Implementing developmentally staged algorithms would require reliable age estimation, raising concerns about privacy and accuracy. At the same time, providing regulated access as an alternative to complete prohibition may reduce incentives for age misreporting, especially if aided by parental oversight. Moreover, commercial platform incentives, which prioritise user engagement and growth, will conflict with developmental optimisation goals. Significant industry change is unlikely to occur without strong external pressure from governing bodies. New legislation, updated regulatory standards and public accountability mechanisms will be required.

Future research should focus on identifying specific mechanisms linking algorithmically curated social platform use to developmental outcomes, and on testing the effectiveness of targeted design interventions. Interdisciplinary collaboration between neuroscience, psychology, and technology design will be essential.

## 5 Conclusions

Adolescent brain development is characterised by non-linear, asynchronous changes that produce shifting windows of vulnerability and opportunity. Current policy approaches, which rely on binary distinctions between permitted and prohibited exposure, are poorly aligned with these dynamics. Social media platforms are not passive technologies but adaptive algorithmic systems that continuously interact with developing neural circuits. Recognising this interaction reframes social media governance from a question of simple access towards one of developmental optimisation. Developmentally staged algorithmic design provides a framework to address this. By appropriately calibrating exposure to the evolving properties of the adolescent brain, it may be possible to reduce risk while amplifying the benefits of healthy digital engagement.
